# RNA-Sequencing Analysis Revealed Genes Associated with Sweet Potato (*Ipomoea batatas* (L.) Lam.) Responses to Stem Rot during Different Infection Stages

**DOI:** 10.3390/genes14122215

**Published:** 2023-12-14

**Authors:** Chen Li, Liang Zhang, Honghu Ji, Weihan Song, Ziyu Zhong, Meiqiao Jiang, Yungang Zhang, Qiang Li, Linrun Cheng, Meng Kou

**Affiliations:** 1 Jinhua Academy of Agricultural Sciences, Jinhua 321000, China; lichen_xz@163.com (C.L.); 18858984660@163.com (L.Z.); hnjihonghu9903@163.com (H.J.); zzyhnky@163.com (Z.Z.); jiangmeiqiao@163.com (M.J.); 2 Xuzhou Institute of Agricultural Sciences in Jiangsu Xuhuai District/Key Laboratory of Biology and Genetic Breeding of Sweetpotato, Ministry of Agriculture and Rural Affairs, Xuzhou 221131, China; xzsongweihan@163.com (W.S.); zhangyungang@jaas.ac.cn (Y.Z.); liqiang@jaas.ac.cn (Q.L.)

**Keywords:** sweet potato, stem rot, transcriptome, gene, transcription factor

## Abstract

The sweet potato, which is an important tuber crop in China, is susceptible to a variety of pathogens and insect pests during cultivation and production. Stem rot is a common sweet potato disease that seriously affects tuber yield and quality. Unfortunately, there have been relatively few studies on the mechanism mediating the stem rot resistance of sweet potatoes. In this study, a transcriptome sequencing analysis was completed using Xushu 48 samples at different stages (T1, T2, and T3) of the stem rot infection. The T1 vs. T2, T1 vs. T3, and T2 vs. T3 comparisons detected 44,839, 81,436, and 61,932 differentially expressed genes (DEGs), respectively. The DEGs encoded proteins primarily involved in alanine, aspartate, and glutamate metabolism (ko00250), carbon fixation in photosynthetic organisms (ko00710), and amino sugar and nucleotide sugar metabolism (ko00520). Furthermore, some candidate genes induced by phytopathogen infections were identified, including gene-encoding receptor-like protein kinases (*RLK5* and *RLK7*), an LRR receptor-like serine/threonine protein kinase (*SERK1*), and transcription factors (*bHLH137*, *ERF9*, *MYB73*, and *NAC053*). The results of this study provide genetic insights that are relevant to future explorations of sweet potato stem rot resistance, while also providing the theoretical basis for breeding sweet potato varieties that are resistant to stem rot and other diseases.

## 1. Introduction

Sweet potatoes, which are widely cultivated in China, have been used as an important food source, feed material, and industrial raw material because they are rich in carbohydrates, nutrients, and functional ingredients [1]. In terms of planting area and output, China is the primary producer of sweet potatoes worldwide. However, the continuous planting of sweet potatoes for many years has increased the prevalence of a variety of diseases. Sweet potato stem rot has recently become one of the most serious sweet potato diseases in various parts of China, including Zhejiang, Guangdong, Hebei, Henan, and other provinces [2,3]. Serious cases of sweet potato stem rot often lead to seedling death and decreases in yield and quality. Moreover, sweet potato stem rot can occur in all life stages, as well as during post-harvest storage. The main symptoms of stem rot are dark brown and waterlogged disease spots on the stem or petiole. As the disease worsens, the stem segment softens and dissociates, ultimately resulting in wilting and death [4].

Sweet potato stem rot was first detected and reported in the United States in 1974 [5]. The causative pathogen was initially identified as *Erwinia chrysanthemi*, but it was later re-classified as a species in the genus *Pectobacterium* according to an analysis of 16S rDNA [6]. The use of new techniques for identifying species resulted in the classification of some stem rot pathogens in the genus *Pectinobacillus*, whereas others were classified in the new genus *Dickeya* [7]. Efforts to identify sweet potato stem rot pathogens started relatively late in China. On the basis of molecular biology and sequencing data, *E. chrysanthemi* and *D. dadantii* were identified as the main pathogens responsible for sweet potato stem rot in China [3,4,8]. More specifically, the pathogen causing sweet potato stem rot in Zhejiang province was identified as *D. dadantii* [9], which can infect more than 60 different host plants, such as ornamental plants (e.g., *Convolvulaceae*, *Compositae*, and *Orchidaceae*) and agricultural plants (e.g., *Solanaceae*, *Leguminosae*, *Gramineae*, *Dioscorea*, and *Cruciferae*), especially important food crops, including sweet potatoes, rice, corn, onions, eggplants, peppers, carrots, and tomatoes [10]. Therefore, molecular plant pathologists consider *D. dadantii* as one of the top 10 phytopathogenic bacteria [11].

Recent serious outbreaks of stem rot in sweet potato-producing areas in China have restricted the development of the sweet potato industry. The disease management strategies used to date (e.g., chemical control and agronomic practices) have been insufficient for controlling sweet potato stem rot. Moreover, there are no sweet potato varieties that are highly resistant or immune to stem rot [9]. Thus, there is an urgent need for developing and cultivating stem rot-resistant sweet potato resources, which largely depends on the identification of disease resistance genes. As a powerful analytical tool, transcriptome sequencing (RNA-seq) technology has been widely used to investigate different plant types. There has been considerable research on the transcriptomes of plants infected with pathogens, which has resulted in the identification of many pathogen-responsive genes as well as the characterization of disease resistance mechanisms. For example, Xiao et al. sequenced the transcriptomes of resistant and susceptible wheat materials, which revealed that *PR5* and *PR14* expression levels are significantly up-regulated in disease-resistant wheat [12]. In another study, an RNA-seq analysis showed that during the lettuce response to an infection with *Botrytis cinerea*, the expression of genes related to phenylpropane and terpenoid synthesis is up-regulated, whereas the photosynthetic pathway is inhibited [13]. A resistance gene (*SWC*) in a *Capsicum* species was identified via RNA-seq, with the encoded protein involved in the perception of the effector *AvrBs4* released by *Xanthomonas* species [14]. The continual enhancement of RNA-seq technology will enable researchers to conduct more detailed and in-depth sequencing analyses of plant transcriptomes in response to pathogen infections. By studying the differential expression of plant genes, we can mine for more reliable candidate genes related to disease resistance and further clarify the molecular mechanism underlying plant disease resistance. Therefore, in this study involving the moderately resistant sweet potato variety Xushu 48, we analyzed the expression of stem rot-responsive genes in different stem rot infection stages. Moreover, candidate sweet potato stem rot resistance genes were identified, which may be relevant to breeding disease-resistant sweet potato varieties.

## 2. Materials and Methods

### 2.1. Plant Materials and Treatments

Xushu 48 is a new stem rot-resistant sweet potato variety selected by the Xuzhou Agricultural Science Research Institute in the Xuhuai region of Jiangsu province, China. Sweet potato stem rot pathogen *D. dadantii* strains Dd1 and Dd2 were collected, isolated, identified, and preserved by the Institute of Plant Protection and Microbiology of the Zhejiang Academy of Agricultural Sciences. The Dd1 and Dd2 mixtures were used to evaluate sweet potato stem rot resistance under laboratory conditions [15]. For both Dd1 and Dd2, a single colony on an NA medium was selected and cultured on fresh NA medium at 28 °C for 36 h. The bacterial mass was rinsed with sterile water and then the bacterial suspension was prepared (10^7^/mL) for the subsequent inoculation of sweet potato samples, as previously described [9].

The resistance of sweet potato seedlings grown in vermiculite was assessed according to the method developed by the Institute of Plant Protection and Microbiology of the Zhejiang Academy of Agricultural Sciences. Briefly, 500 mL of Guangkou tissue culture bottles were filled with vermiculite moistened with sterile water. The stems of seedlings with 3–4 leaves were wounded with sandpaper (5 cm from the stem base), after which the seedlings were placed in vermiculite so that part of the wound site was exposed to the air. The wounded site of each sweet potato stem was inoculated with 1 mL of bacterial suspension, while the control was treated with sterile water instead of bacterial suspension. The same wounded plants were used in the control group and the treatment group, and the other operations were consistent with those in the treatment group except for aseptic water infection in the control group. The inoculated seedlings were incubated in a temperature- and light-controlled culture room set at 28 °C and 80% relative humidity. The inoculation was completed with three biological replicates, each comprising 10 seedlings (one seedling per tissue culture bottle). Disease incidence was monitored daily. According to the previous experimental basis (the data were not published), it was found that sweet potato stem rot began to occur after 3 days of inoculation in some disease-resistant varieties, and waterlogged disease spots appeared. When it reached 6 days, the inoculated stem segment blackened seriously and the plants began to die. Therefore, the following three sample types were collected for the subsequent analysis: infected with sterile water for 0 days (T1), infected for 3 days (T2), and infected for 6 days (T3).

### 2.2. RNA Extraction, Library Preparation for Transcriptome Analysis

Using a Trizol reagent kit (Invitrogen, Carlsbad, CA, USA), total RNA was extracted from the stems of Xushu 48. An Agilent 2100 Bioanalyzer (Agilent Technologies, Palo Alto, CA, USA) and RNase-free agarose gel electrophoresis were used to measure the quality of the RNA. Oligo (dT) beads were used to enrich eukaryotic mRNA following the extraction of total RNA, and the Ribo-ZeroTM Magnetic Kit (Epicentre, Madison, WI, USA) was used to remove rRNA from prokaryotic mRNA. Then, the enriched mRNA fragments were cut into short segments using a fragment buffer and reverse-transcribed into cDNA using random primers. Second-strand cDNA was synthesized by utilizing DNA polymerase I, RNase H, dNTP, and buffer. Next, the cDNA fragments were purified using the QiaQuick PCR extraction kit (Qiagen, Venlo, The Netherlands), followed by end repair, the addition of PolyA, and connection to the Illumina sequencing adapter. The agarose gel electrophoresis method was used to screen according to the size of the ligation products, and the Illumina HiSeq 2500 from Gene Denovo Biotechnology Co., (Guangzhou, China) was used for amplification and sequencing.

### 2.3. Transcriptome Assembly and Screening of DEGs (Differentially Expressed Genes)

Transcriptome sequencing was completed based on NGS and 3GS, and TPM, FPKM, RPKM, and fold changes (Fc) for each repeat of each library were recorded. The sequences obtained from NGS and 3GS were aligned, and similar sequence data from all libraries/samples were gathered. The transcriptome data were compared and annotated with the reported sweet potato genome database (http://sweetpotato.uga.edu/, accessed on 18 June 2023).

A DEG analysis was performed between the two groups using the DESeq2 software version 1.20.0 [16], while EdgeR version 3.38.1 [17] was used for DEG analysis between the two samples. When the false discovery rate (FDR) of a gene/transcript is ≤0.05 and the absolute folding change is ≥2, it is confirmed as a significantly differentially expressed gene/transcript [18]. 

### 2.4. Gene Functional Annotation and KEGG (Kyoto Encyclopedia of Genes and Genomes) Enrichment Analysis

Gene functions were annotated based on the following databases: NR (https://www.ncbi.nlm.nih.gov/, accessed on 17 August 2023), NT (https://www.ncbi.nlm.nih.gov/, accessed on 17 August 2023), Pfam (http://pfam.sanger.ac.uk/, accessed on 17 August 2023), KOG/COG (https://www.ncbi.nlm.nih.gov/cog/, accessed on 17 August 2023), and SWISS-PROT (http://www.ebi.ac.uk/uniprot/, accessed on 17 August 2023). The differentially expressed genes were analyzed via GO [19] annotation and KEGG [20] pathway enrichment analysis using the GOSeqR software package version 1.22.0 and KEGG Orthology software version 4.2.

### 2.5. qRT-PCR (Real-Time Quantitative PCR) Validation

Thirteen randomly selected unigenes were included in the quantitative real-time polymerase chain reaction (qRT-PCR) analysis, which was performed using the QuantStudio™ 6 Flex Real-Time PCR System (Thermo Fisher Scientific, Waltham, MA, USA). Total RNA was extracted from sweet potato stems using the Total RNA Rapid Extraction kit (Shanghai Generay Biotech Co., Ltd., Shanghai, China) and then reverse-transcribed to cDNA using the ReverTra Ace^®^ qPCR RT Master Mix with a gDNA Remover Kit (FSQ-301, Toyobo Co., Ltd., Osaka, Japan). The qRT-PCR analysis was conducted using the SYBR Green Real Time PCR Master Mix (10 μL), forward/reverse primers (10 μM, 0.5 μL), a cDNA template (1 μL), and ddH_2_O (8 μL), with *ARF* (JX177359) serving as the reference gene [21]. The qRT-PCR primers for each unigene were designed using Primer3Plus (Supplementary Table S1). The relative expression levels for three independent experiments were calculated using the 2^−ΔΔCt^ method [22].

## 3. Results

### 3.1. Phenotypic Analysis of Inoculated Sweet Potato Seedlings

**Figure 1:**
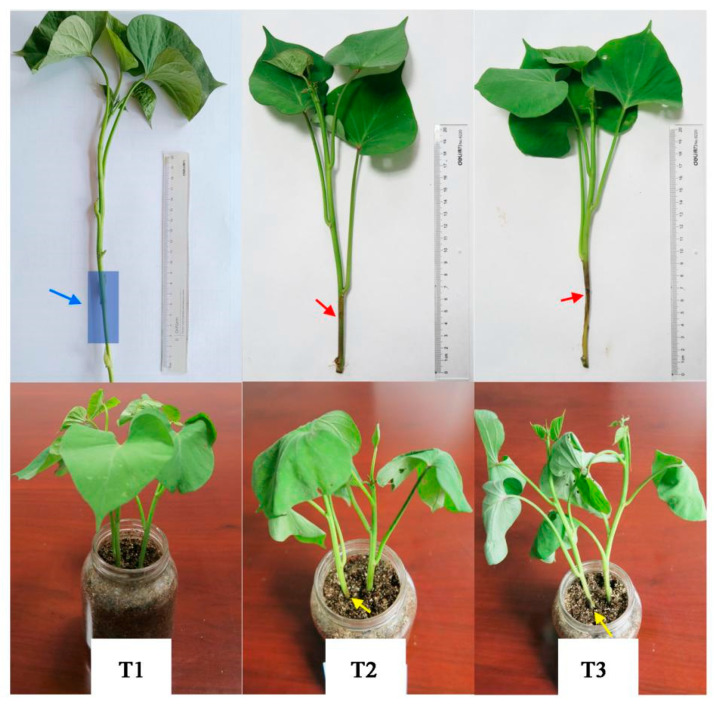
Xushu 48 seedling phenotypes at different stages of the stem rot infection. The red arrow indicates the stem lesion on the sweet potato seedling infected with stem rot. T1, T2, and T3 refer to the seedlings infected with sterile water for 0 days, the seedlings infected with stem rot for 3 days, and the seedlings infected with stem rot for 6 days, respectively. The blue area pointed to by the blue arrow in the picture is the wound area from sand wear. The yellow arrow indicates the location of the inoculated bacterial suspension. The red arrow indicates the epidemic area of stem rot.

The phenotypic changes in the healthy Xushu 48 seedlings inoculated with the isolated sweet potato stem rot pathogen were examined (Figure 1). The stems and leaves of the control (i.e., uninoculated) sweet potato seedlings were green, with no black spots, and the leaves were extended normally. At 3 days post-inoculation, the seedlings had a black–brown stem lesion and leaves that started to droop. At 6 days post-inoculation, the stem was rotted and the leaves were severely drooping. Moreover, the seedlings were beginning to die.

### 3.2. Transcriptome Analysis

**Figure 2:**
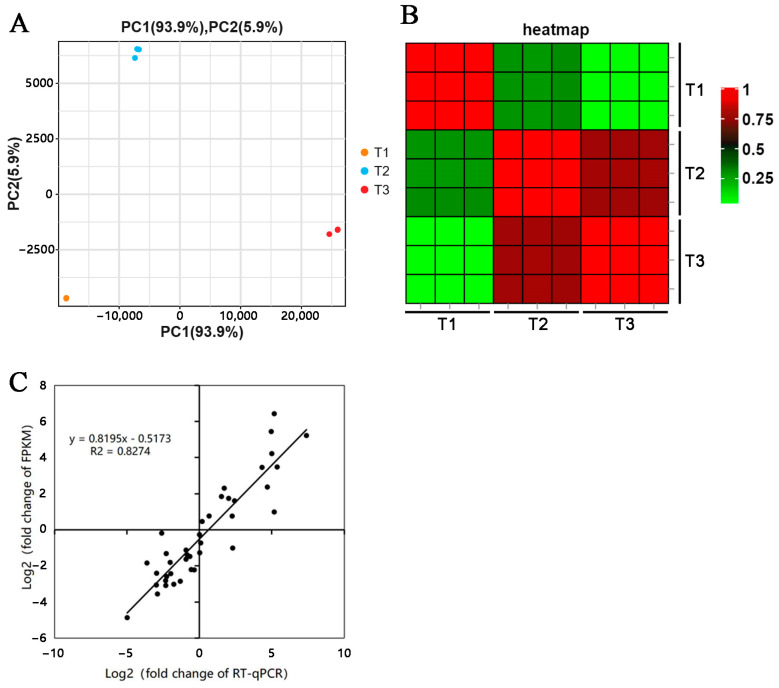
Analysis of the relationships among the transcriptome samples and verification of the expression data via qRT-PCR. Principal component (**A**) and heat map (**B**) analyses of the stem rot pathogen-infected Xushu 48 stem segments collected at different infection stages. (**C**) Correlation analysis of the transcriptome data and the qRT-PCR data.

The principal component and heat map analyses of the stem rot pathogen-infected Xushu 48 samples, collected in three stages (T1, T2, and T3), revealed that the T1, T2, and T3 samples were clearly separated, whereas the replicates in each stage were clustered (Figure 2A,B). These results reflected the stability and reliability of the transcriptome data. Thirteen DEGs were selected for the qRT-PCR analysis. The transcriptome data (fold-change in the FPKM values) were closely correlated with the qRT-PCR data for the 13 DEGs (R^2^ = 0.826). Accordingly, the transcriptome data were accurate and reliable. For each sample, more than 6 GB of sequencing data was generated (>99% clean data). Thus, the amount of sequencing data satisfied the study requirements (Supplementary Table S2).

### 3.3. Identification and Analysis of DEGs

**Figure 3:**
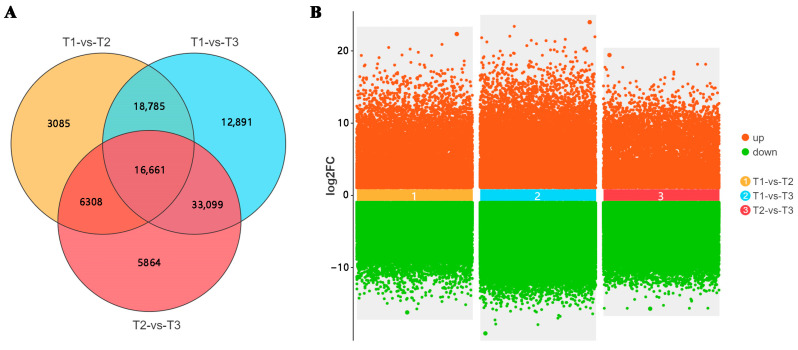
Total number of differentially expressed genes (DEGs) in stem rot pathogen-infected Xushu 48 stem segments collected at different stages. (**A**) Venn diagram of DEGs. The number in each circle represents the number of DEGs in the corresponding group. The number of common DEGs between groups is provided in the overlapping regions. (**B**) Multi-point difference scatter diagrams of the comparisons. Red and green dots represent up-regulated and down-regulated DEGs, respectively.

The DEGs in the stem rot pathogen-infected Xushu 48 samples, collected at different stages, were analyzed. A total of 44,839 DEGs were detected between T2 and T1, including 17,351 up-regulated genes and 27,488 down-regulated genes. There were 81,436 DEGs between T3 and T1, including 16,820 up-regulated genes and 64,616 down-regulated genes. There were 61,932 DEGs between T3 and T2, including 11,369 up-regulated genes and 50,563 down-regulated genes (Figure 3).

### 3.4. Enriched GO Terms and KEGG Pathways

**Figure 4:**
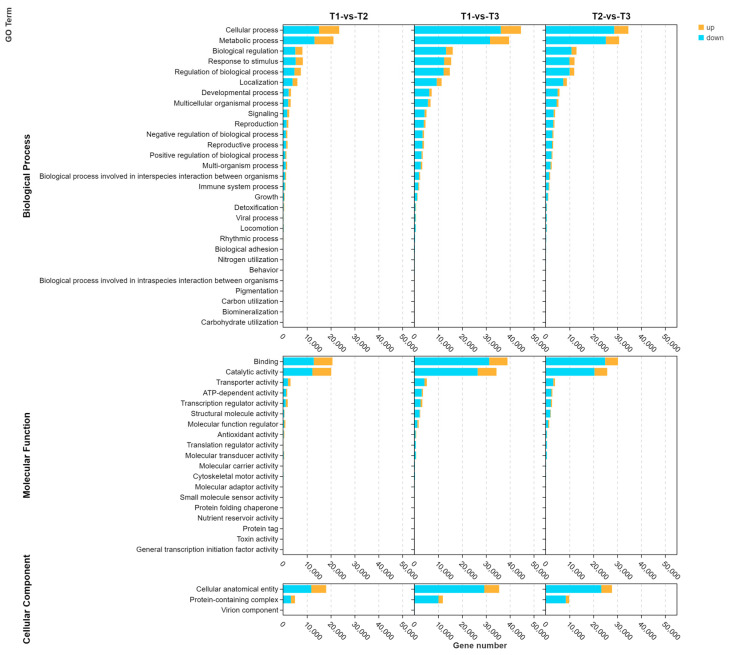
Enriched GO terms assigned to the DEGs in the stem rot pathogen-infected Xushu 48 stem segments at different stages. Orange and blue represent the up-regulated and down-regulated DEGs, respectively.

**Figure 5:**
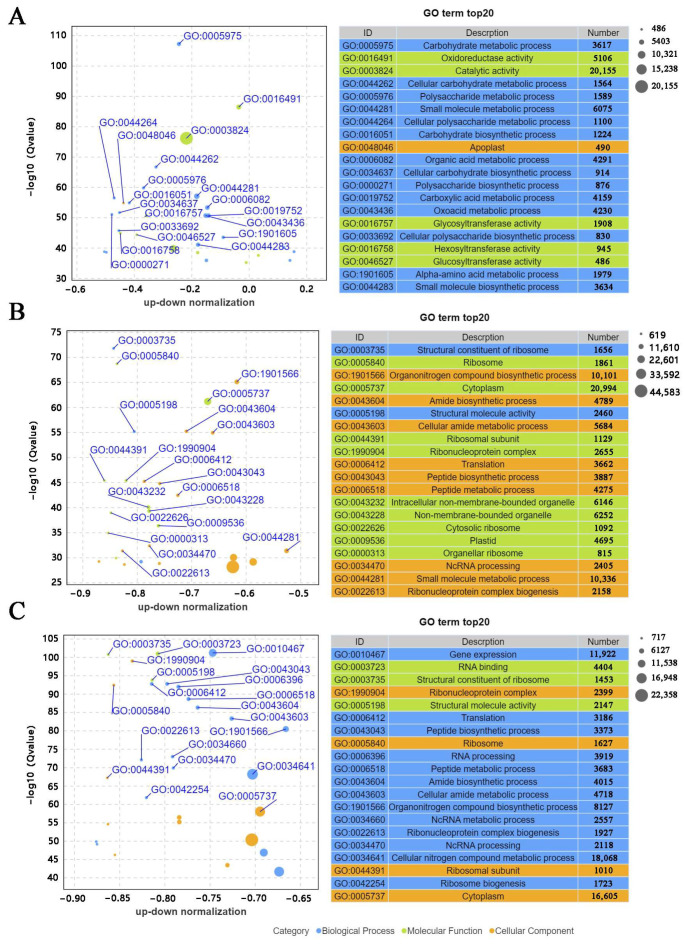
Top 20 significantly enriched GO terms. (**A**) Top 20 significantly enriched GO terms assigned to the DEGs between T1 and T2. (**B**) Top 20 significantly enriched GO terms assigned to the DEGs between T1 and T3. (**C**) Top 20 significantly enriched GO terms assigned to the DEGs between T2 and T3.

The five main enriched GO terms assigned to the DEGs in the three analyzed stages (T1, T2, and T3) were cellular process, metabolic process, binding, catalytic activity, and cellular anatomical entity. From T1 to T3, the number of DEGs annotated with the five main GO terms initially increased and then decreased. Hence, T2 may be an important period for resistance to the stem rot pathogen (Figure 4). The top 20 significantly enriched GO terms assigned to the DEGs between T1 and T2 included catalytic activity (GO:0016194), oxidoreductase activity (GO:0003824), and small-molecule metabolic process (GO:0044281). As the infection duration increased (i.e., comparison between T1 and T3), the main enriched GO terms among the DEGs were cytoplasm (GO:00005737), organonitrogen compound biosynthetic process (GO:1901566), and small-molecule metabolic process (GO:0044281), which were assigned to 20,994, 10,101, and 16,605 DEGs, respectively. For the comparison between T2 and T3, the DEGs were mostly annotated with cytoplasm (GO:00005737), organonitrogen compound biosynthetic process (GO:1901566), and cellular nitrogen metabolic process (GO:0034641) (Figure 5). 

**Figure 6:**
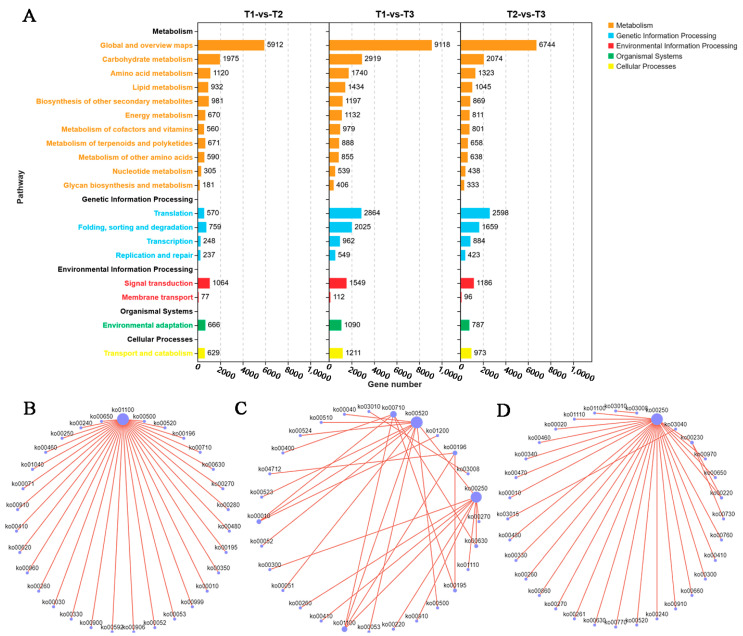
Enriched KEGG pathways among the DEGs in the stem rot pathogen-infected Xushu 48 stem segments at different stages. (**A**) Quantitative statistics of the DEGs associated with different metabolic pathways. The numbers next to the orange, blue, red, green, and yellow bars represent the number of DEGs associated with metabolism, genetic information processing, environmental information processing, organismal systems, and cellular processes, respectively. Correlations among the metabolic pathways associated with the DEGs between T1 and T2 (**B**), T1 and T3 (**C**), and T2 and T3 (**D**). Each purple dot in (**B**–**D**) represents a metabolic pathway, and the lines connecting the two points indicate that there is an upstream and downstream relationship between the two metabolic pathways or that there are common differentially expressed genes.

The main enriched KEGG pathways among the DEGs in the three stages were global and overview maps, carbohydrate metabolism, transcription, signal transduction, and other biological pathways (Figure 6A). The analysis of the associations between the enriched metabolic pathways among the DEGs between T1 and T2 indicated that metabolic pathway ko01100 was associated with other metabolic pathways, including glycolysis/gluconeogenesis (ko00010), carbon fixation in photosynthetic organisms (ko00710)/amino sugar and nucleotide sugar metabolism (ko00520), and fructose and mannose metabolism (ko00051) (Figure 6B). For the T1 vs. T3 comparison, the following three enriched metabolic pathways were the main nodes connected with other metabolic pathways: alanine, aspartate, and glutamate metabolism (ko00250); carbon fixation in photosynthetic organisms (ko00710); and amino sugar and nucleotide sugar metabolism (ko00520) (Figure 6C). For the comparison between T2 and T3, alanine, aspartate, and glutamate metabolism (ko00250) was the main node, which was associated with amino sugar and nucleotide sugar metabolism (ko00520) and glycolysis/gluconeogenesis (ko00010) (Figure 6D). These results suggest that carbohydrate metabolism may be critical for the stem rot resistance of sweet potatoes.

### 3.5. Analysis of the DEG Expression Trends

**Figure 7:**
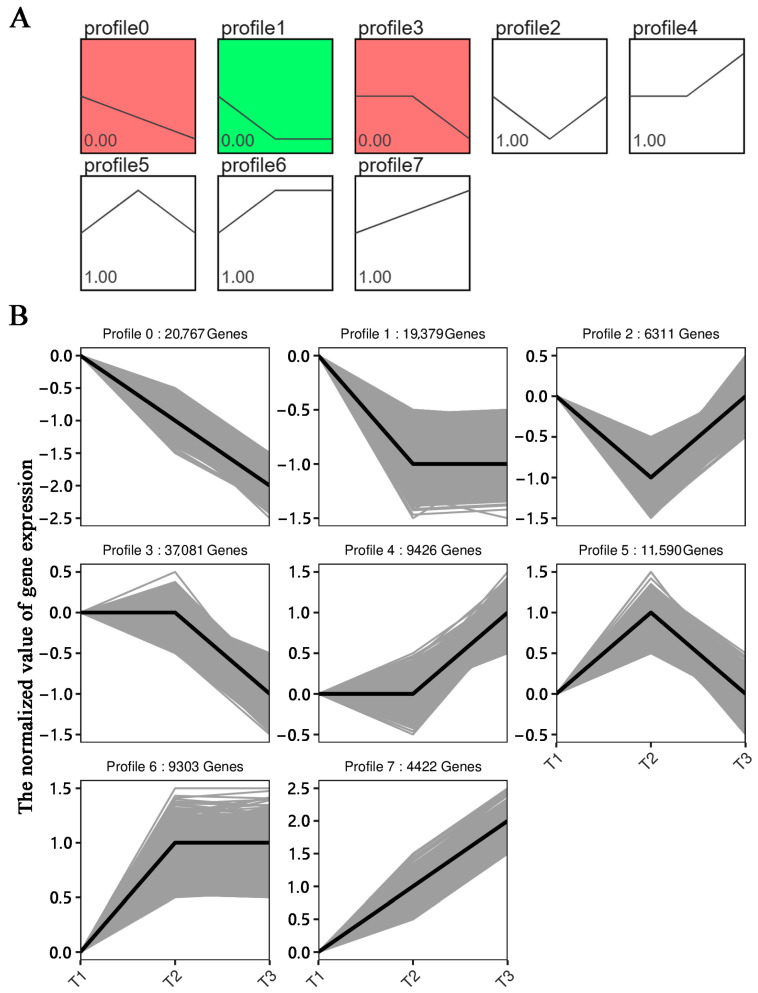
Analysis of the DEG expression trends in the stem rot pathogen-infected Xushu 48 stem segments at different stages. (**A**) Analysis of the significance of the DEGs in each module. (**B**) Number of DEGs in each module. The black lines in the figure show the changing trend of gene expression corresponding to each module at different stages. Each gray line in the picture represents a DEG. The Y axis represents the normalized value of gene expression at different infection time points, with positive number being up-regulated and negative number being down-regulated.

The DEG expression trends in the three stages were grouped into eight modules. Increases in the duration of the stem rot infection may lead to increases or decreases in the expression of genes responsive to stem rot. Therefore, we focused on the DEGs in modules 0 and 7. There were 20,767 DEGs in module 0 and 4422 DEGs in module 7 (Figure 7). Many of the genes in these two modules encode proteins related to responses to infections and defenses against plant pathogens, including the receptor-like protein kinases *RLK5* (Ibat.Brg.05B_G013520) and *RLK7* (Ibat.Brg.08C_G006740), the LRR receptor-like serine/threonine protein kinase *SERK1* (Ibat.Brg.06B_G025950), and the serine/threonine protein kinase 11-interacting protein-like *STK11IP* (Ibat.Brg.10A_G016990). Thirteen plant disease resistance-related genes were selected for a qRT-PCR analysis of their relative expression levels. The qRT-PCR data were consistent with the transcriptome data (Supplementary Table S3).

### 3.6. Analysis of Differentially Expressed Transcription Factors

**Figure 8:**
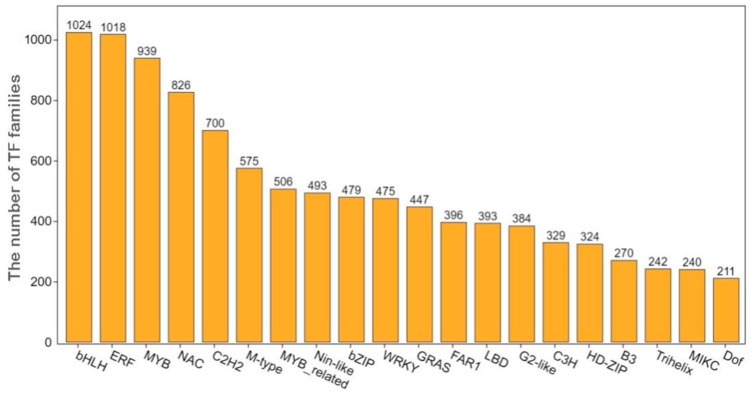
Quantitative analysis of differentially expressed transcription factors. The number above each column represents the number of transcription factor transcripts from that particular family.

The transcription factor-encoding genes among the DEGs were analyzed. Many DEGs encoding the bHLH, ERF, MYB, NAC, and C2H2 transcription factors were identified. Specifically, 1024 transcripts of genes in the bHLH transcription factor family and 1018 transcripts of genes in the ERF transcription factor family were detected. Additionally, 939, 826, and 700 transcripts of genes encoding MYB, NAC, and C2H2 transcription factor family members were identified, respectively (Figure 8). The bHLH, ERF, MYB, NAC, and C2H2 transcription factors accounted for 34.75% of all of the identified transcription factors, implying that they may be related to the sweet potato defense response to stem rot. Members of the bHLH, ERF, and MYB transcription factor families are important for plant growth and development, as well as for responses to biotic and abiotic stresses. According to the transcriptome analysis, the bHLH family members *bHLH137* (Ibat.Brg.04D_G021250) and *bHLH162* (Ibat.Brg.06B_G028170), the ERF family members *ERF9* (Ibat.Brg.09A_G006830) and *ERF4* (Ibat.Brg.10F_G018750), and the MYB family members *MYB73* (Ibat.Brg.01F_G031560) and *MYB2* (Ibat.Brg.02A_G015020) may help to protect sweet potatoes from stem rot infections. The NAC family members are plant-specific transcription factors that function as key regulators of plant stress resistance. A total of 820 NAC transcription factor transcripts were identified in the transcriptome, including the transcripts of a number of significantly up-regulated and down-regulated genes, including *NAC053* (Ibat.Brg.02B_G005540), *NAC078* (Ibat.Brg.02F_G003820), *NAC081* (Ibat.Brg.03E_G007680), and *NAC021* (Ibat.Brg.04D_G028260).

## 4. Discussion

Like other crops, sweet potatoes are susceptible to various pathogens that can adversely affect cultivation, including the stem rot pathogen, which has detrimental effects on sweet potato yield and nutritional quality. Sweet potato stem rot was first discovered and reported in the United States in 1974 [5]. The pathogen responsible for sweet potato stem rot was subsequently isolated and identified as *D. dadantii* [2]. However, there has been limited research on the mechanism underlying the stem rot resistance of sweet potatoes. There are few reports on the mining of stem rot resistance genes in sweet potatoes. Therefore, in this study, the transcriptome of Xushu 48, which is moderately resistant to stem rot, was analyzed during different stem rot infection stages. The results showed that after the sweet potatoes were infected with pathogens of stem rot, the redox, catalytic activity, and other related genes were induced and the autoimmune system was activated to resist the infection of pathogens.

Plants rely on the innate immune system to perceive potential pathogens and limit their harmful effects. Plant innate immunity is mediated by the following two related systems: pathogen-associated molecular pattern-triggered immunity (PTI) and effector-triggered immunity (ETI). More specifically, PTI represents the first layer of the plant immune system [23]. Most pathogens are recognized by transmembrane pattern recognition receptors (PRRs). After perceiving the pathogen, PRRs transmit signals through several proteins, including Botrytis-induced kinase 1 (*BIK1*) [24], mitogen-activated protein kinases (*MAPKs*), and calcium-dependent protein kinases (*CDPKs*) [25], thereby activating appropriate immune responses (e.g., the accumulation of reactive oxygen species and callose). In the current study, an RNA-seq analysis revealed DEGs encoding *MAPKs* and *CDPKs,* including *MAPK20* (Ibat.Brg.05A_G019550), *MAPK18* (Ibat.Brg.06E_G014790), and *CPK1* (Ibat.Brg.01E_G026440). In addition, the main enriched GO terms assigned to the DEGs during the early stage of the stem rot infection were catalytic activity (GO:0016194) and oxidoreductase activity (GO:0003824), whereas the enriched GO terms among the DEGs during the later infection stage were mainly the organonitrogen compound biosynthetic process (GO:1901566) and the small-molecule metabolic process (GO:0044281). The expression patterns of these DEGs were in accordance with the biological processes activated in resistant plants in response to infections by phytopathogens.

Because they are continuously evolving, some phytopathogens produce effectors that enable them to overcome the PTI of plants. However, ETI evolved as a second layer of the plant immune system [26,27]. Plants also evolved specific resistance (R) genes, most of which encode a nucleotide-binding leucine-rich repeat (*NLR*) receptor. These *NLR* receptors can directly or indirectly detect toxic proteins in cells and trigger a series of immune responses, ultimately leading to disease resistance. The transcriptome analysis conducted in the current study revealed many DEGs related to leucine-rich repeats and their related regulatory kinases, including the LRR receptor-like serine/threonine protein kinase-encoding gene *FEI1* (Ibat.Brg.01D_G026840) and the plant intracellular Ras-group-related LRR protein-encoding gene *PIRL2* (Ibat.Brg.07D_G004510). These candidate genes may be important for regulating sweet potato resistance to the stem rot pathogen.

Plant transcription factors have crucial functions related to the resistance to pathogens. The WRKY transcription factors are the most prominent transcription factors involved in molecular pattern-triggered immunity. Specifically, 15 WRKY transcription factors are strongly induced, including *WRKY18*, *WRKY33*, and *WRKY40* [28]. These three WRKY transcription factors are also considered to be important nodes in the WRKY regulatory network [29]. A total of 475 WRKY transcripts were detected in the transcriptome, including the transcripts of *WRKY33* (Ibat.Brg.15B_G007050) and *WRKY40* (Ibat.Brg.S022350). Furthermore, bHLH [30], ERF [31], NAC [32,33], MYB [34], and other transcription factor families are also reportedly related to plant resistance to pathogens or other biotic stressors. Several additional transcription factors, such as *bHLH137* (Ibat.Brg.04D_G021250), *bHLH162* (Ibat.Brg.06B_G028170), *ERF9* (Ibat.Brg.09A_G006830), *ERF4* (Ibat.Brg.10F_G018750), *MYB73* (Ibat.Brg.01F_G031560), and *MYB2* (Ibat.Brg.02A_G015020), were also detected. These candidate transcription factors may be useful for studying the molecular mechanism underlying the stem rot resistance of sweet potatoes.

## 5. Conclusions

In this study, an RNA-seq analysis was performed using Xushu 48 sweet potato stem samples collected at different stem rot infection stages (T1, T2, and T3). The subsequent comparisons detected 44,839 (T1 vs. T2), 81,436 (T1 vs. T3), and 61,932 (T2 vs. T3) DEGs. These DEGs were mainly associated with alanine, aspartate, and glutamate metabolism (ko00250); carbon fixation in photosynthetic organisms (ko00710); and amino sugar and nucleotide sugar metabolism (ko00520). Moreover, some candidate genes related to plant responses to pathogen infections, such as receptor-like protein kinase genes (*RLK5* and *RLK7*), the LRR receptor-like serine/threonine protein kinase gene *SERK1*, and transcription factor genes (*bHLH137*, *ERF9*, *MYB73*, and *NAC053*), were identified. These study findings may be relevant to future investigations on the genetic basis of the stem rot resistance of sweet potatoes, with potential implications for breeding disease-resistant sweet potato varieties.

## Data Availability

Data are contained within the article and Supplementary Materials.

## Supplementary Materials

The following supporting information can be downloaded at https://www.mdpi.com/article/10.3390/genes14122215/s1: Table S1: Primer sequences; Table S2: Filtered data; Table S3: Comparison of qRT-PCR and transcriptome data.
