# Supplementary material for: RNA-Sequencing Analysis Revealed Genes Associated with Sweet Potato (Ipomoea batatas (L.) Lam.) Responses to Stem Rot during Different Infection Stages
Source: Genes (Basel). 2023 Dec 14;14(12):2215. doi: 10.3390/genes14122215 (PMC10742929; doi:10.3390/genes14122215)
Supplement: Supplementary file 1 [file genes-14-02215-s001.zip › genes-2728089-supplementary.pdf]

Supplementary Table

Table S1 Primer sequence

Genes ID	Symbol	Primer sequence
Ibat.Brg.01D_G026840	*IbFEI1*	AGTACATGCAAAGTGGCCGA<br>TGCCTGCACTCCTTCACAAT
Ibat.Brg.02E_G006670	*IbLRR-kinase*	CCTGGGAATTGCGAGAGGTT<br>GCCACGCATTGCATACTCAG
Ibat.Brg.07D_G004510	*IbPIRL2*	AATCTGCAATGCCGCGAATC<br>TATTCTCTTCCGCCGCCATC
Ibat.Brg.13C_G011370	*IbIRL5*	CTGCCACCAAGACCTTCCAT<br>TCAACAACACCGCCTCTCTC
Ibat.Brg.03A_G009250	*IbSERK4*	TCAGGGCGGTTTTGGTAAGG<br>GGGTATACCAGAAGGCGCTC
Ibat.Brg.05F_G002900	*IbGLR2.5*	TGGGATGGCGTTTCATGTGA<br>CCTGAGTTTCTCGAACCCCC
Ibat.Brg.05B_G030500	*IbSNL6*	AGAATGTCCTAGAGGCATGTGC<br>ATTTCCCACGCTGCCTTTTC
Ibat.Brg.03B_G004180	*IbMAPKKK20*	GTGTTGTTGGAGTTCGCTTCTC<br>GCCAATCCGAAATCCCCAATC
Ibat.Brg.02C_G017680	*IbStk16*	GGATGGAACCTTGCTGGACA<br>ATTGCAAGAGGTGGTAGCCC
Ibat.Brg.06E_G021150	*IbSTK38L*	ATGGAATGCGATTGGTGGTC<br>CCCAACCTTGATTCCACATCAC
Ibat.Brg.01F_G030190	*IbRLK7*	CTTGCCCAATGGAAGCTTATGG<br>TTTGCAAGGCCGAAATCTGC
Ibat.Brg.15B_G024350	*IbRLK5*	AATTTGAGCGGCGAAATCCC<br>TTGCCGAAAACCTCAAAGGC
Ibat.Brg.13C_G025850	*IbRLP4*	AGGCAAGAGGGTGTTTGATG<br>AGATCAGCTGGCACAATAGC
Ibat.Brg.01D_G026840	*IbFEI1*	AGTACATGCAAAGTGGCCGA<br>TGCCTGCACTCCTTCACAAT
JX177359	*IbARF*	CTTTGCCAAGAAGGAGATGC<br>TCTTGTCCTGACCACCAACA

Table S2 Data filtering statistical table

Sample	Raw Data	Clean Data (%)	Adapter (%)	Low Quality (%)	Poly A (%)	N (%)
T1-1	68647334	68270444 (99.45%)	27398 (0.04%)	349296 (0.51%)	0 (0.00%)	196 (0.00%)
T1-2	76133694	75724958 (99.46%)	36120 (0.05%)	371532 (0.49%)	0 (0.00%)	1084 (0.00%)
T1-3	72375724	72001774 (99.48%)	28154 (0.04%)	345616 (0.48%)	0 (0.00%)	180 (0.00%)
T2-1	67369710	66845856 (99.22%)	81710 (0.12%)	442110 (0.66%)	0 (0.00%)	34 (0.00%)
T2-2	69275988	68765074 (99.26%)	72490 (0.10%)	438148 (0.63%)	0 (0.00%)	276 (0.00%)
T2-3	69334552	68799308 (99.23%)	80984 (0.12%)	453944 (0.65%)	0 (0.00%)	316 (0.00%)
T3-1	67691814	67165224 (99.22%)	77444 (0.11%)	449106 (0.66%)	0 (0.00%)	40 (0.00%)
T3-2	69340112	68763092 (99.17%)	77270 (0.11%)	499408 (0.72%)	0 (0.00%)	342 (0.00%)
T3-3	64391870	63856990 (99.17%)	106688 (0.17%)	427604 (0.66%)	0 (0.00%)	588 (0.00%)

Note: RawDatas: original number of reads offline; CleanData (%): number of high-quality reads and percentage (based on RawReads); Adapter (%): number of reads containing adapter and percentage (based on RawReads); LowQuality (%): low-quality reads, that is, number of reads with more than 50% base mass value $Q \leq 20$ in single-ended read and (based on RawReads) percentage; polyA (%): number of polyA reads and (based on RawReads) percentage. N (%): number of highly filtered reads with N ratio and (based on RawReads) percentage.

Table S3 Comparison of q-PCR and transcriptome data

ID	Symbol	Treatment	q-PCR		FPKM	
			Mean value	SD	Mean value	SD
Ibat.Brg.01D_G026840	FEI1	T1	0.032	0.004	13.645	0.862
		T2	0.008	0.001	3.903	0.285
		T3	0.001	0.001	0.469	0.255
Ibat.Brg.02E_G006670	LRR-Kinase	T1	0.044	0.001	26.504	0.505
		T2	0.023	0.008	8.573	0.326
		T3	0.013	0.004	3.278	0.549
Ibat.Brg.07D_G004510	RIRL2	T1	0.005	0.005	2.661	0.406
		T2	0.099	0.030	29.203	1.964
		T3	0.159	0.004	49.566	3.761
Ibat.Brg.13C_G011370	IRL5	T1	0.000	0.000	0.199	0.199
		T2	0.009	0.004	8.632	0.903
		T3	0.011	0.007	17.113	2.204
Ibat.Brg.03A_G009250	SERK4	T1	0.006	0.001	6.902	0.437
		T2	0.017	0.006	24.756	0.952
		T3	0.020	0.003	34.066	1.268
Ibat.Brg.05F_G002900	GLR2.5	T1	0.000	0.000	0.512	0.099
		T2	0.015	0.005	5.707	0.502
		T3	0.062	0.011	19.068	1.216
Ibat.Brg.05B_G030500	SNL6	T1	0.001	0.001	14.016	0.576
		T2	0.006	0.007	42.713	2.992
		T3	0.029	0.009	72.330	5.978
Ibat.Brg.03B_G004180	MAPKKK20	T1	0.060	0.008	43.837	2.391
		T2	0.015	0.002	8.152	0.934
		T3	0.008	0.002	3.736	0.592
Ibat.Brg.02C_G017680	STK16	T1	0.018	0.004	21.750	1.204
		T2	0.001	0.001	6.082	0.313
		T3	0.007	0.004	3.018	0.732
Ibat.Brg.06E_G021150	STK38L	T1	0.061	0.018	35.147	1.080
		T2	0.065	0.003	21.235	2.632
		T3	0.041	0.005	7.634	1.004
Ibat.Brg.01F_G030190	RLK7	T1	0.063	0.015	23.027	0.373
		T2	0.064	0.012	19.119	0.240
		T3	0.013	0.001	2.716	0.274
Ibat.Brg.15B_G024350	PLK5	T1	0.025	0.008	12.159	1.755
		T2	0.004	0.000	10.699	1.186
		T3	0.003	0.005	2.296	0.696
Ibat.Brg.13C_G025850	RLP4	T1	0.005	0.002	8.899	0.430
		T2	0.001	0.000	3.575	0.243
		T3	0.001	0.000	1.481	0.071
